# RM2Target: a comprehensive database for targets of writers, erasers and readers of RNA modifications

**DOI:** 10.1093/nar/gkac945

**Published:** 2022-10-27

**Authors:** Xiaoqiong Bao, Yin Zhang, Huiqin Li, Yuyan Teng, Lixia Ma, Zhihang Chen, Xiaotong Luo, Jian Zheng, An Zhao, Jian Ren, Zhixiang Zuo

**Affiliations:** State Key Laboratory of Oncology in South China, Cancer Center, Collaborative Innovation Center for Cancer Medicine, School of Life Sciences, Sun Yat-sen University, Guangzhou 510060, China; State Key Laboratory of Oncology in South China, Cancer Center, Collaborative Innovation Center for Cancer Medicine, School of Life Sciences, Sun Yat-sen University, Guangzhou 510060, China; State Key Laboratory of Oncology in South China, Cancer Center, Collaborative Innovation Center for Cancer Medicine, School of Life Sciences, Sun Yat-sen University, Guangzhou 510060, China; State Key Laboratory of Oncology in South China, Cancer Center, Collaborative Innovation Center for Cancer Medicine, School of Life Sciences, Sun Yat-sen University, Guangzhou 510060, China; State Key Laboratory of Oncology in South China, Cancer Center, Collaborative Innovation Center for Cancer Medicine, School of Life Sciences, Sun Yat-sen University, Guangzhou 510060, China; State Key Laboratory of Oncology in South China, Cancer Center, Collaborative Innovation Center for Cancer Medicine, School of Life Sciences, Sun Yat-sen University, Guangzhou 510060, China; State Key Laboratory of Oncology in South China, Cancer Center, Collaborative Innovation Center for Cancer Medicine, School of Life Sciences, Sun Yat-sen University, Guangzhou 510060, China; State Key Laboratory of Oncology in South China, Cancer Center, Collaborative Innovation Center for Cancer Medicine, School of Life Sciences, Sun Yat-sen University, Guangzhou 510060, China; Experimental Research Center, Cancer Hospital of the University of Chinese Academy of Sciences (Zhejiang Cancer Hospital), Hangzhou 310016, China; State Key Laboratory of Oncology in South China, Cancer Center, Collaborative Innovation Center for Cancer Medicine, School of Life Sciences, Sun Yat-sen University, Guangzhou 510060, China; State Key Laboratory of Oncology in South China, Cancer Center, Collaborative Innovation Center for Cancer Medicine, School of Life Sciences, Sun Yat-sen University, Guangzhou 510060, China

## Abstract

RNA modification is a dynamic and reversible process regulated by a series of writers, erasers and readers (WERs). Abnormal changes of WERs will disrupt the RNA modification homeostasis of their target genes, leading to the dysregulation of RNA metabolisms such as RNA stability and translation, and consequently to diseases such as cancer. A public repository hosting the regulatory relationships between WERs and their target genes will help in understanding the roles of RNA modifications in various physiological and pathological conditions. Previously, we developed a database named ‘m6A2Target’ to host targets of WERs in m6A, one of the most prevalent RNA modifications in eukaryotic cells. To host all RNA modification (RM)-related WER–target associations, we hereby present an updated database, named ‘RM2Target’ (http://rm2target.canceromics.org/). In this update, RM2Target encompasses 1 619 653 WER–target associations for nine RNA modifications in human and mouse, including m6A, m6Am, m5C, m5U, m1A, m7G, pseudouridine, 2′-O-Me and A-to-I. Extensive annotations of target genes are available in RM2Target, including but not limited to basic gene information, RNA modifications, RNA–RNA/RNA–protein interactions and related diseases. Altogether, we expect that RM2Target will facilitate further downstream functional and mechanistic studies in the field of RNA modification research.

## INTRODUCTION

RNA modifications have been known to play critical roles in modulating RNA metabolisms, such as gene transcription, RNA stability, RNA splicing, nuclear localization and translation (1). RNA modifications are dynamically mediated by three different classes of proteins: writers, erasers and readers (WERs). Writers can regulate the deposition of RNA modifications; e.g., METTL3 and NSUN2 can write N6-methyladenosine (m6A) and 5-methylcytosine (m5C) to their target genes, respectively (2,3). Erasers remove RNA modifications from target genes; e.g., FTO is an eraser for m6A and ALKBH3 for N1-methyladenosine (m1A) (4,5). Readers serve their functions by recognizing RNA modification sites in target genes; e.g., YTHDF1/2/3 are readers for m6A (6) and ALYREF is a reader for m5C (7). The dysregulation of WERs has been proved to be associated with various diseases, including cancer, cardiovascular diseases and neurological diseases (8–10). The same WER may have distinct functions under different conditions. For example, METTL3 plays an oncogenic role in most cancer types (11,12), but it has also been reported to have tumor-suppressive functions in certain cancer types, such as kidney cancer (13). This is mainly because perturbation of a WER may selectively affect different sets of target genes in different conditions. Taken together, identifying WER–target associations is particularly important for studying the functions and regulatory mechanisms of RNA modifications in various physiological and pathological conditions. However, there is yet no public repository to host WER–target associations across different RNA modifications.

Benefitting from the development of experimental and high-throughput sequencing technologies, more evidence could be provided to explore the relationship between WER and target genes. For instance, immunoprecipitation (14) and certain next-generation sequencing technologies such as RIP-seq (15) and CLIP-seq (16) can directly and effectively elucidate the binding relationship between WER proteins and target RNAs. Perturbation techniques such as small interfering RNA (siRNA) (17) and CRISPR/Cas9 system (18) together with high-throughput sequencing could be used to systematically evaluate the perturbation effects of WERs on the RNA metabolisms of specific genes. In 2020, we developed m6A2Target, a comprehensive resource for targets of m6A writers, erasers and readers (19). m6A2Target is the first resource focused on the WER–target associations and has been widely used since its publication. Over the past 2 years, there has been an explosion of studies related to RNA modifications. With the discovery of more WERs, the number of target genes has also expanded, necessitating an update of m6A2Target.

**Figure 1.**
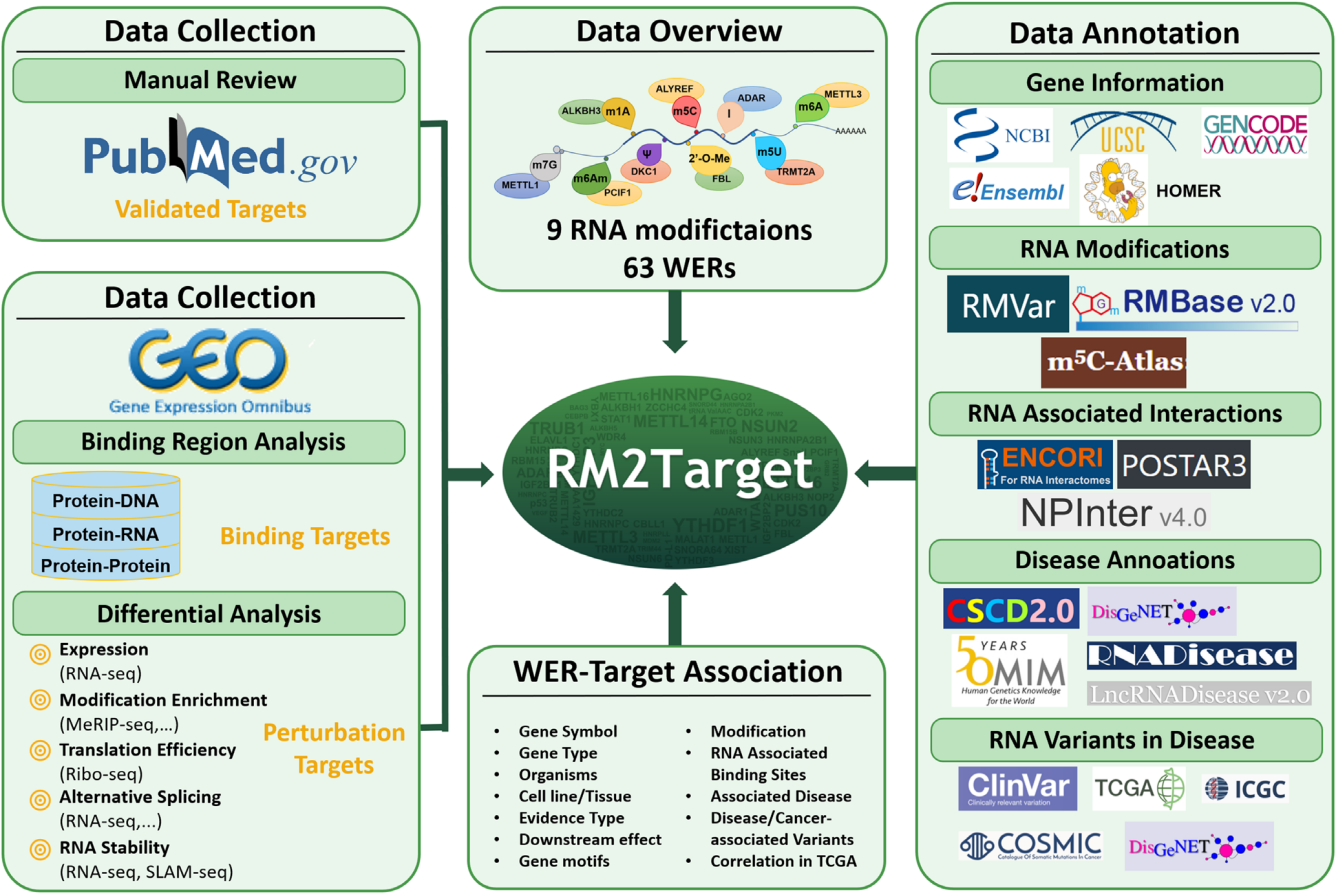
Overall design and construction of RM2Target. WER–target associations deposited in RM2Target were categorized into three evidence types, namely ‘validated targets’ derived from manual curation, ‘binding targets’ inferred from binding region analysis and ‘perturbation targets’ predicted from differential analysis. RM2Target provides basic gene information as well as abundant annotations, including RNA modifications, RNA–RNA/RNA–protein interactions, expression correlation between WERs and their target genes in TCGA cancer types, and RNA–disease associations.

In this study, we present an updated database called RM2Target (http://rm2target.canceromics.org/) to host WER–target associations for 63 WERs from two organisms and nine types of RNA modifications, including N6-methyladenosine (m6A), N6,2′-*O*-dimethyladenosine (m6Am), N1-methyladenosine (m1A), pseudouridine (ψ), 5-methyluridine (m5U), 5-methylcytosine (m5C), 7-methylguanosine (m7G), 2′-*O*-methylation and A-to-I RNA editing. Compared to m6A2Target, RM2Target is far superior in all aspects (Supplementary Table S1). RM2Target encompasses 1 619 653 WER–target associations in different cell lines or tissue types derived from published literature and public high-throughput datasets. All records are categorized into three evidence types, namely ‘validated targets’ collected from low-throughput methods, ‘binding targets’ inferred from high-throughput methods such as iCLIP-seq, eCLIp-seq, HITS-CLIP-seq, PAR-CLIP-seq, RIP-seq and ChIP-seq and ‘perturbation targets’ inferred from WERs perturbation followed by high-throughput sequencings such as RNA-seq, MeRIP-seq and Ribo-seq (Figure 1). To allow users to further study the functions of WERs and target genes and investigate the relationship between RNA modifications and diseases, RM2Target provides basic gene information as well as abundant annotations, including RNA modifications, RNA–RNA/RNA–protein interactions, expression correlation between WERs and their target genes in TCGA cancer types, and RNA–disease associations. Overall, we expect that RM2Target will be a valuable resource for researchers who are aiming at exploring the regulatory principles of WERs or finding certain therapeutic targets.

## MATERIALS AND METHODS

### Data collection and quality control

RM2Target comprises a manually curated catalog of 63 reported WERs, including 60 WERs for human (35 writers, seven erasers, 18 readers), and 36 WERs for mouse (18 writers, three erasers, 15 readers) (Supplementary Table S2). All related literature and high-throughput datasets were collected from NCBI PubMed and Gene Expression Omnibus (GEO) (20) by keywords combinations ‘(WER gene symbol [Title/Abstract]) OR (RNA modification type [Title/Abstract])’. Based on the different data source, RM2Target categorized all WER–target associations into three types: (i) ‘Validated’: we retrieved a total of 8468 papers published up to April 2022. By manual curation, 927 articles providing direct experimental evidence were strictly screened out (Supplementary Table S3). All experimental details and downstream effects were integrated manually. (ii) ‘Binding’: 72 datasets about protein–DNA interactions, protein–RNA interactions and protein–protein interactions from ChIP-seq, RIP-seq, CLIP-seq and mass spectrometry were collected. Further analyses were performed to identify the binding targets of different WERs in different cell lines or tissues. (iii) ‘Perturbation’: 285 datasets were collected under different perturbation conditions, such as knock-out, overexpression and mutation. Different analyses were performed to obtain potential targets changed at the level of gene expression, modification, translation, alternative splicing or stability according to the sequencing method, like RNA-seq, MeRIP-seq or Ribo-seq (Supplementary Table S4).

For all datasets, Genome Reference Consortium Human Build 38 (hg38) and Genome Reference Consortium Mouse Build 39 (mm39) were used as the reference genomes for human and mouse, respectively. fastp (21) was employed to perform quality control and preprocessing of all raw data.

### Derivation of potential targets with binding evidence

To obtain potential targets that have protein–protein interactions with WERs, mass spectrometry results were summarized from the supplementary data of relevant literature.

To obtain potential targets that have protein–DNA interactions with WERs, preprocessed ChIP-seq data were aligned to the reference genome using bowtie2 (22), and peaks were called using MACS2 (23). HOMER (24) was used to annotate peaks to genes. All genes with ChIP-seq peaks were considered as potential WER targets with protein–DNA binding evidence.

To obtain potential targets that have protein–RNA interactions with WERs, RIP-seq and various types of CLIP-seq data were collected. For RIP-seq, STAR (25) was used for read alignment and then RSEM (26) was used to obtain the count and FPKM matrix. Genes with a fold change between IP and INPUT greater than 2 (IP/INPUT > 2) were considered as potential binding targets. For various types of CLIP-seq, bowtie2 (22) was first invoked to do the read mapping. Then, CLIP Tool Kit (27) was used to identify the potential binding sites for iCLIP-seq, eClip-seq and HITS-CLIP-seq, and PARalyzer (28) was used for PAR-CLIP-seq. HOMER was used to annotate all binding sites. All genes with binding sites were considered as potential WER targets with protein–RNA binding evidence.

### Derivation of potential targets with perturbation evidence

To obtain potential targets with perturbation evidence, differential analyses were performed between control group versus perturbed group at five levels: RNA expression, modification, translation, alternative splicing and stability. Group information for all analyses can be found in the Supplementary Table S3.

For RNA expression, preprocessed RNA-seq data were aligned to the reference genome using STAR (25). RSEM (26) was then used to generate gene counts, and DESeq2 (29) was used to obtain the differentially expressed genes (*P*-value < 0.05, fold change > 1.5). For modification enrichment, MeRIPseqPipe (30) was used to automatically analyze MeRIP-seq data, where MACS2 and MeTPeak (31) were used for m6A peak calling and DESeq2 was set to compare the modification levels (*P*-value < 0.05, fold change > 1.5). For m5C BS-seq, information about the modification sites and quantification results were downloaded from relevant GEO datasets. For RNA stability, half-life of mRNA was calculated according to a previously published paper (32). Genes with a fold change in half-life between perturbed group and control group > 1.2 were considered as potential targets. For translation efficiency, Ribo-seq data were filtered for mitochondrial DNA and ribosomal RNA using bowtie2 (22), followed by read alignment using STAR (25). RSEM (26) was used to obtain the count and FPKM matrix of genes. The difference between two groups was calculated using log_2_(FPKM of perturbed group/FPKM of control group), with a cutoff of 1.5 for the fold change. For alternative splicing, rMATS (33) was used to detect different alternative splicing events. The differential alternative splicing events (FDR < 0.01) were considered as potential targets.

### Motif analysis of target genes

To identify the consensus motifs of target genes for each WER, we use the top 200 most reliable targets determined by confidence score to perform motif analysis with HOMER (24). Only targets with binding peaks detected by CLIP-seq or modification peaks detected by MeRIP-seq among top 200 targets will be involved in this analysis. Related peak clusters were set as the target sequences, and a set of background clusters was generated with shuffleBed program from BEDTools (34) to randomly shuffle regions of the same size as the clusters throughout the gene regions. The parameter of motif length was set as 5, 6, 7,8. Top 10 significant motifs (*P*-value < 0.01) were used and displayed in the web page.

### Annotation of WER–target associations

Basic information of WERs and target genes, such as official gene symbol, gene ID, gene type and genome location, were preferentially extracted from GENCODE (35) annotation files (human: v40, mouse: vM29). Ensembl (36), UCSC (37) and GtRNAdb (38) databases were used for information supplementation. Deprecated or substituted versions of genes were filtered out. Experimental details and sample information were obtained from the source literature or relevant datasets.

To better illustrate the WER–target associations, modification sites in RMVar (39), RMBase 2.0 (40) and m5C-Atlas (41) were collected to predict the role of RNA modifications in the regulatory relationship between WERs and targets. ENCORI (42), POSTAR3 (43) and NPinter4.0 (44) were used to predict the RNA–RNA or RNA–protein interactions of target genes to provide supplementary evidence of WER–target associations. Additionally, to explore the relationship between targets and diseases, TCGA (45) gene expression data were integrated into RM2target, allowing users to investigate the correlation between the expression of WERs and their targets in any cancer type of interest. Furthermore, RNA–disease associations with experimental evidence were collected from OMIM (46), DisGeNET (47), RNADisease (48), LncRNADisease2.0 (49) and CSCD2.0 (50). For disease-associated variants, data were collated from DisGeNET (47) and ClinVar (51), and for cancer-associated variants, from COSMIC (52), ICGC (53) and TCGA (45). These annotations may deliver new insights into the underlying pathogenesis from the perspective of WER–target associations. To unify all results, the genomic coordinates of all data resources were further converted to hg38 or mm39 using the LiftOver program (37). The gene symbols were also converted using the standard procedures.

### Database and web interface implementation

MySQL was used to store and manage all data in RM2Target. The server-backend development was based on java and the web-frontend interfaces were implemented in Hyper Text Markup Language (HTML), Cascading Style Sheets (CSS) and JavaScript (JS). All the interactive diagrams were generated by ECharts to visualize the analysis results. Furthermore, RM2Target implemented a genome browser to present genomic annotations using UCSC Genome Browser (https://genome.ucsc.edu/) (37).

## RESULTS

### Database content

**Figure 2.**
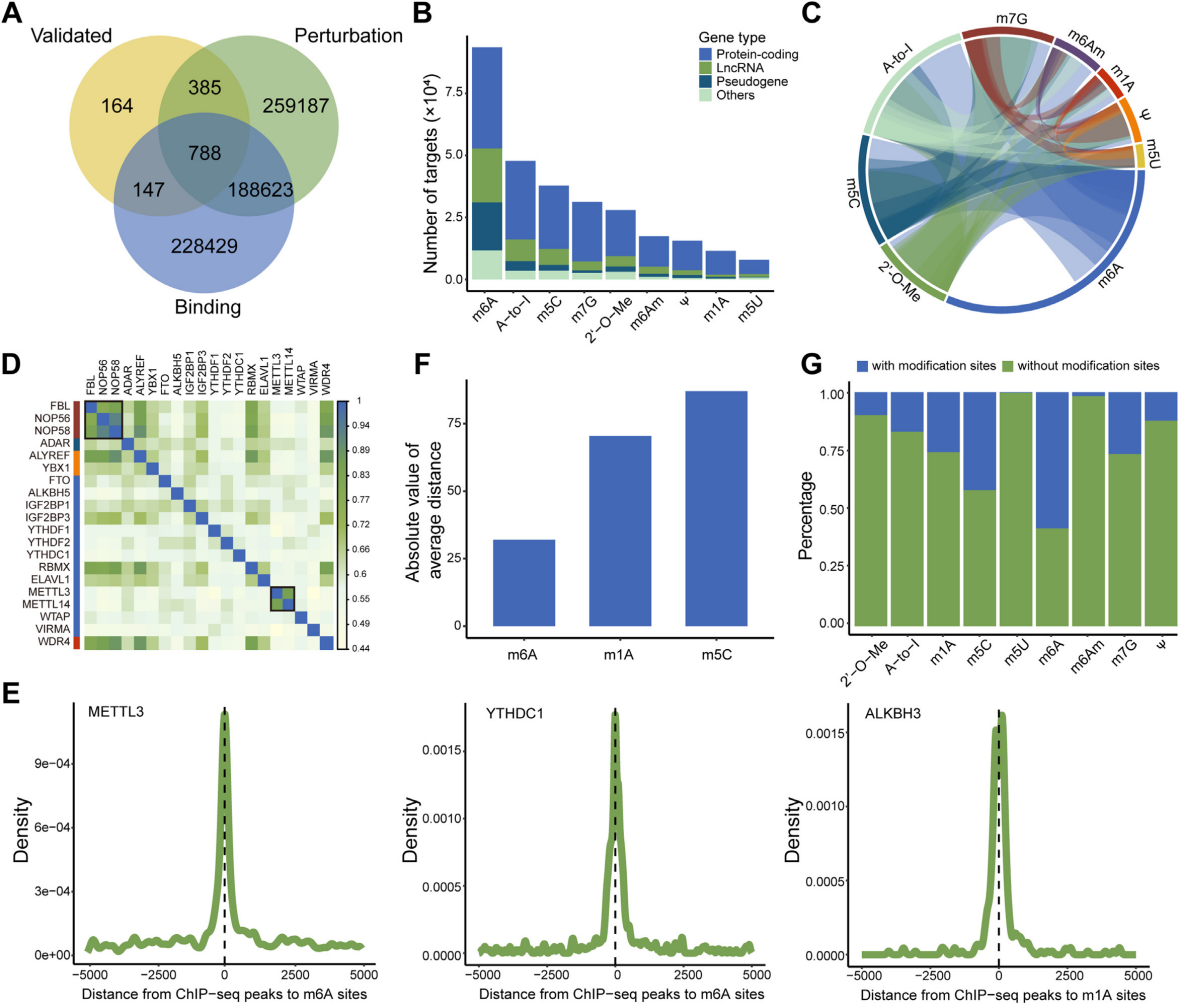
Overview of WER–target associations in RM2Target. (**A**) The overlap between records with different evidence types among the shared WERs. (**B**) The gene type distribution of all target genes for WERs from different modifications. Others present those low-frequency gene types, such as tRNA, snoRNA. (**C**) Circular plot displays the associations between WERs from different modifications. Links refer to the number of target genes in the intersection. (**D**) Sharing target genes between WERs. The top 20 WERs with the most target genes are shown. The overlap degree of target genes between two WERs is calculated using the ratio of intersection and union. (**E**) The density of distance from the center of ChIP-seq peaks to the nearest modification sites. (**F**) The average distance from the center of ChIP-seq peaks to the nearest modification sites of m6A, m1A, m5C. (**G**) The proportion of target genes with or without corresponding modifications.

Currently, RM2Target includes a total of 1 619 653 WER–target associations covering 63 WERs and nine RNA modifications from human and mouse (Table 1). Three evidence types of WER–target associations were deposited in RM2Target: (i) ‘Validated’: 1530 and 584 WER–target associations for human and mouse were validated by *in vivo* or *in vitro* experiments using western blot, RT-qPCR, RNA stability assay, RIP assay, luciferase reporter assay, etc. methods, respectively. (ii) ‘Binding’: 461 746 and 61 395 WER–target associations with binding evidence were predicted by high-throughput analyses for human and mouse, respectively. Among them, there were 451 016 WER–target associations with protein–RNA interactions for human and 55 004 for mouse. A total of 3100 WER–target associations with protein–protein interactions were recorded in human. Growing studies have demonstrated that WERs may localize to the different region of chromatin and subsequently affect the deposition of RNA modifications co-transcriptionally (54–56). Therefore, RM2Target also included 7630 and 6391 WER–target associations with protein–DNA interactions in human and mouse, respectively, which may uncover another layer of gene expression regulation from the perspective of the transcription processes. (iii) ‘Perturbation’: 646 539 and 447 859 WER–target associations were inferred from WERs perturbation for human and mouse, respectively. Previous studies have shown that RNA modifications play vital roles in certain fundamental biological processes, such as mRNA stability (57), splicing (58) and translation (59). Consequently, RM2Target performed differential analyses at five levels, namely expression, modification, translation, stability and alternative splicing. For human, there were 339 604 WER–target associations with RNA level changes, 60 176 with modification level changes, 117 866 with translation efficiency changes, 27 700 with mRNA stability changes and 101 193 with differential alternative splicing events. For mouse, there were 243 043 WER–target associations with RNA level changes, 38 107 with modification level changes, 90 067 with translation efficiency changes, 31 084 with mRNA stability changes and 45 558 with differential alternative splicing events. We observed a highly significant proportion of 89% of WER–target associations validated either by ‘Binding’ or ‘Perturbation’ evidence, indicating the reliability of WER–target associations in RM2Target (Figure 2A). Due to the analysis strategy of high-throughput sequencing data, the target genes are mainly mRNAs and lncRNAs (Figure 2B).

**Table 1.** WER–target associations with different evidence types in RM2Target

		*Homo sapiens*									*Mus musculus*								
			Binding			Perturbation						Binding			Perturbation				
Modification type	WER type	Validated	Protein–protein	protein–DNA	Protein–RNA	Expression	AS	Translation	stability	modification	Validated	Protein–protein	protein–DNA	Protein–RNA	Expression	AS	Translation	Stability	Modification
2′-O-Me	Writer	8	0	0	71 539	6 144	1 276	6 330	0	0	1	0	0	0	0	0	0	0	0
A-to-I	Writer	111	0	0	27 955	21 238	12 956	3 805	0	0	24	0	0	0	21 232	5 568	0	0	0
m1A	Writer	2	0	0	0	0	0	0	0	0	0	0	0	0	0	0	0	0	0
	Eraser	10	0	783	0	1 864	2 103	0	0	0	2	0	0	0	7 047	121	0	0	0
m5C	Writer	21	0	0	0	6 881	1 515	0	0	4 074	6	0	0	0	0	0	0	0	0
	Eraser	9	0	378	0	1 864	2 103	0	0	0	2	0	0	0	7 047	121	0	0	0
	Reader	61	0	5 835	39 335	13 452	4 043	0	0	0	29	0	2 998	0	5 487	1 323	0	0	0
m5U	Writer	3	0	0	7 972	0	0	0	0	0	1	0	0	0	0	0	0	0	0
m6A	Writer	449	1 948	515	72 051	98 088	20 920	36 910	12 649	42 245	176	0	2 297	9 419	86 916	18 216	35 224	10 195	25 679
	Eraser	172	0	0	19 607	42 347	10 898	11 912	8 962	8 735	94	0	0	0	22 433	2 349	10 259	0	1 465
	Reader	497	1 152	119	162 273	108 517	35 136	38 559	6 092	854	150	0	1 096	45 585	67 256	13 738	27 226	20 889	10 963
m6Am	Writer	3	0	0	0	2 062	441	1 037	0	0	1	0	0	0	11 110	2 087	2 818	0	0
	Eraser	105	0	0	12 394	21 177	5 277	5 327	0	4 268	84	0	0	0	13 854	1 995	7 703	0	0
	Reader	5	0	0	0	0	0	0	0	0	1	0	0	0	0	0	0	0	0
m7G	Writer	21	0	0	29 914	957	27	13 564	0	0	5	0	0	0	661	40	6 837	0	0
Pseudouridine	Writer	53	0	0	7 976	15 013	4 498	422	0	0	8	0	0	0	0	0	0	0	0

### Complex regulatory network among WERs and target genes revealed by RM2Target

#### Crosstalk between different WERs

The writers, erasers and readers of the same RNA modification often co-operate to control cellular phenotypes. For example, a recent study showed that the collaboration of a set of WERs of m6A (‘writer: METTL14’, ‘eraser: ALKBH5’ and ‘reader: YTHDF3’) regulates cancer growth and progression (60). Moreover, increasing evidence indicates a frequent crosstalk among different RNA modifications. For example, NSUN2-mediated m5C modifications can promote METTL3-mediated m6A modifications, and vice versa (61). m6A modifications will inhibit A-to-I editing level probably by blocking the binding of ADAR and target genes (62). We systematically analyzed the data collected in RM2Target to explore the crosstalk between different WERs across different RNA modifications. We observed intensive crosslinks between target genes of WERs from different modifications (Figure 2C). As expected, the target genes of m6A writers METTL3 and METTL14 have a high degree of overlap (Figure 2D), consistent with the fact that METTL3 and METTL14 often function as a heterodimer complex. Additionally, FBL, NOP56 and NOP58, writers of 2′-*O*-methylation, which are proved to act as the core proteins of box C/D small nucleolar ribonucleoprotein complexes (snoRNPs) (63), also share similar sets of target genes.

#### Crosstalk between RNA modifications and gene transcription

The deposition of RNA modifications is usually considered as a co-transcriptional process (54–56). More recently, increasing studies revealed that RNA modifications can also regulate gene transcription (64,65). By integrated analysis of peak sites from the ChIP-seq data of WERs and RNA modification sites, we found that the ChIP-seq peak sites of m6A WER, such as METTL3, YTHDC1, were close to corresponding m6A sites, and the peaks sites of m1A WERs, such as ALKBH3 was close to corresponding m1A sites (Figure 2E), suggesting a strong crosstalk between these RNA modifications and gene transcription. We calculated the average distance between the ChIP-seq peak center of WER and nearest RNA modification sites and considered this average distance indicated the crosstalk between RNA modification and gene transcription. As a result, we observed that m6A modification is mostly involved in the crosstalk and m5C is least involved in the crosstalk (Figure 2F).

#### RNA modification independent WER–target associations

Recent studies showed that some known WERs of RNA modification have modification-independent functions. For instance, the m6A writer METTL3 could promote the translation of target gene PAPBC1 without m6A modification (66). The m6A writer METTL16 exerts an m6A-independent function to facilitate translation and tumorigenesis (67). We systematically investigated the RNA modification dependent and independent WER–target associations using RM2Target data. We found that only 30% target genes have corresponding RNA modification sites from public databases such as RMbase and RMVar (Figure 2G), which suggests that WERs might have RNA modification independent functions. However, the possibility that the current methods for the detection of RNA modifications may miss many RNA modification sites for various reasons cannot be ignored.

### Web interface and usage

Compared to m6ATarget, the web interface of RM2Target has been redesigned to allow users to browse, search, and download all available RNA modification WER and target gene information more conveniently.

**Figure 3.**
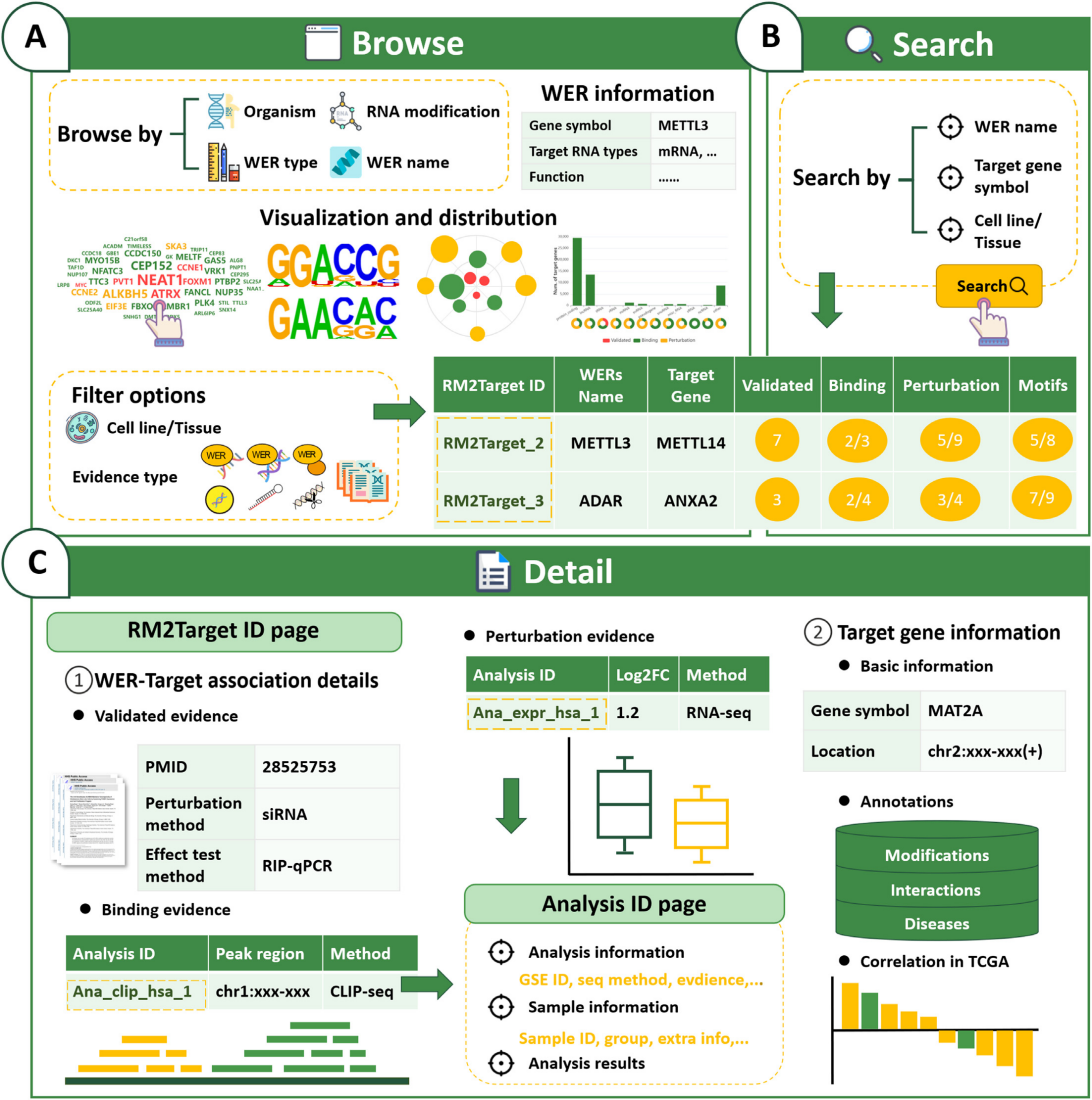
Basic functions of RM2Target web interface. (**A**) The browsing interface of RM2Target. (**B**) The main modules of search interface in RM2Target. (**C**) The detailed information about WER–target associations can be accessed by clicking on the RM2Target ID. Clicking on the analysis ID on the RM2Target ID page links to more details about the specific analysis.

*Browse and search*, in the browse page (Figure 3A), users can browse WER–target associations by WERs of interest. Firstly, the basic information for the selected WER was provided. A word cloud map was then provided to visualize the target genes of the selected WER. The size of each WER was determined by the confidence score, which was defined as the sum of supported literature/dataset number of the three evidence types. Motifs can be used to uncover the biological rules of binding targets. To identify the consensus motif of individual WERs, we use the top 200 most reliable targets determined by confidence score as described above to perform motif analysis. The identified consensus motifs of selected WER were shown in the ‘Browse’ page. As expected, the classical m6A motif ‘RRACH’ (R represents A or G, and H represents A, C or U) was detected in the corresponding m6A writers, erasers and readers in both human and mouse (Supplementary Figure S1A, B), and 77–99% of the targets contain the corresponding motifs (Supplementary Figure S1C). A browse table was provided to show the target genes of selected WER. We provide advanced filter functions in the browse table for users to further obtain more specific queries by combining conditions. Moreover, to help the user to filter the targets from noises, we provide confidence score, number of validated, binding and perturbation evidence, and motif status in the browse table. The targets were sorted by the confidence score. In the search page (Figure 3B), users can search WER–target gene associations by WER name, target gene name and cell line/tissue types.

*Detail*, by clicking on the RM2Target ID in the browse or search result table, a detail page containing the detail information of the WER–target association in a cell line/tissue type was shown. In the detail page, the details of different evidence types including ‘Validated’, ‘Binding’ and ‘Perturbation’ were provided (Figure 3C). In the ‘Validated evidence’ section, literature sources, cell line/tissue information, experimental methods, and the effect of WER on target genes are provided. In the ‘Binding evidence’ section, the binding type such as ‘protein–RNA’ and ‘protein–DNA’, sequencing method, annotation of binding sites, and links to source data were provided. In the ‘Perturbation evidence’ section, the direction of WER perturbation and perturbation effects on target genes at the level of gene expression, translation, modification, stability and alternative splicing were provided. In addition, RM2Target provides many annotations for the target genes, including RNA modification sites, gene associated interactions and related diseases. Besides, the information of gene expression correlation between WER and target gene in cancers calculated from TCGA data were also integrated. By clicking on the analysis ID in the detail page, users can get the information on dataset information, analysis parameters, sample information, and analysis results.

RM2Target allows users to download all validated and potential targets in human and mouse. Detailed guidance on the usage of RM2Target can be found on the ‘Help’ page.

### Application of RM2Target in cancer studies

**Figure 4.**
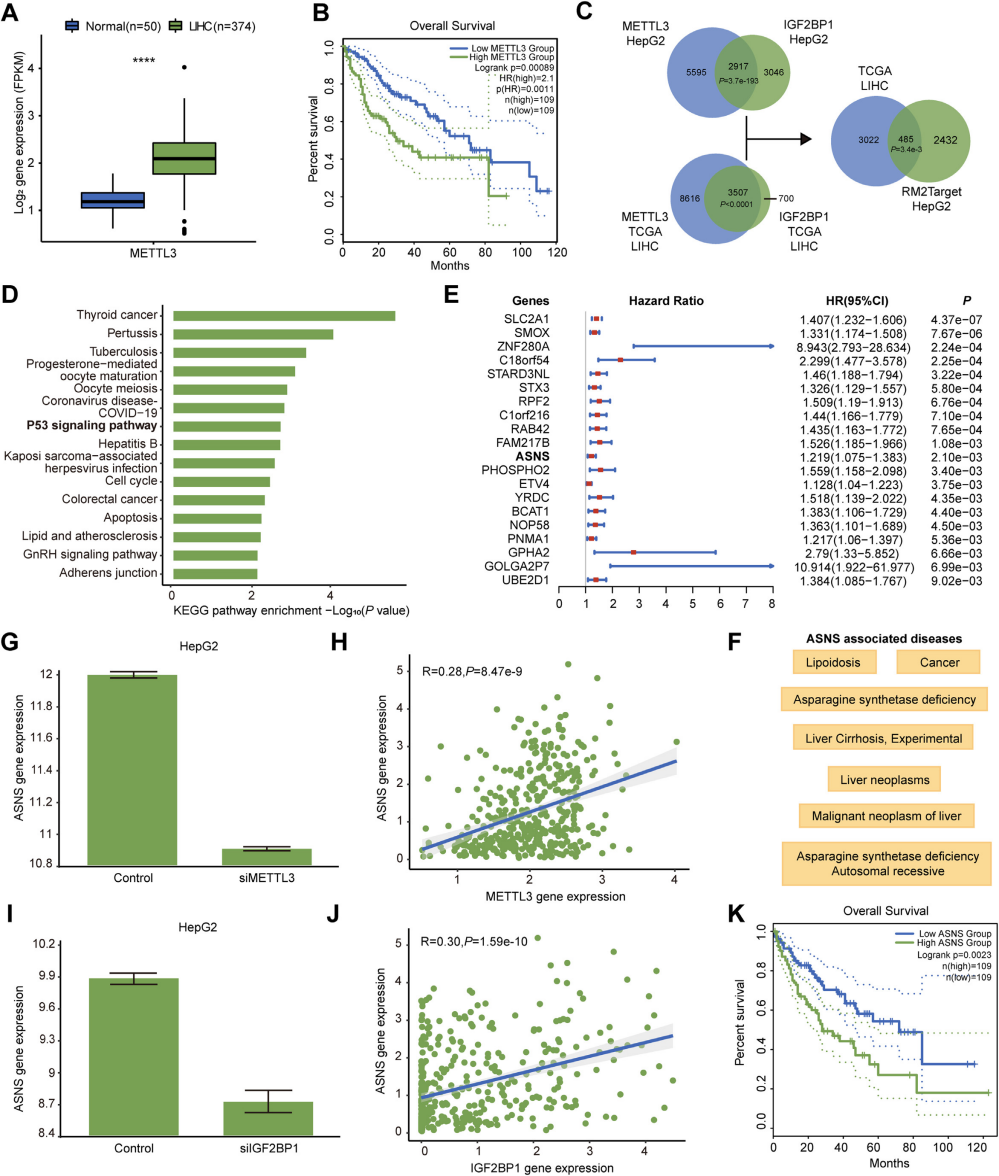
Application of RM2Target. (**A**) The expression difference of METTL3 between tumor and normal tissues in LIHC according to TCGA data. ****P < 0.0001. (**B**) Kaplan–Meier survival curves show the overall survival difference between LIHC patients with high and low METTL3 mRNA expression. (**C**) Venn plots show the overlap between the target genes of METTL3 and IGF2BP1 from HepG2 in RM2Target and the genes significantly associated with both METTL3 and IGF2BP1 in LIHC (|*R*| > 0.25, *P* < 0.05). (**D**) Enrichment of KEGG pathways for the 485 candidate target genes as shown in (C). The top15 significant pathways are displayed. (**E**) Forest plot of the hazard ratio (HR) and 95% confidence intervals (CIs) for the association of target gene expression with overall survival. Top 20 genes are shown. (**F**) Diseases associated with ASNS obtained from the annotation information of RM2Target. (**G**) The expression correlation between METTL3 and ASNS in HepG2 obtained from RM2Target. (**H**) The expression correlation between METTL3 and ASNS in TCGA LIHC data. (**I**) The expression correlation between IGF2BP1 and ASNS in HepG2 obtained from RM2Target. (**J**) The expression correlation between IGF2BP1 and ASNS in TCGA LIHC data. (**K**) Kaplan–Meier survival curves show the overall survival difference between LIHC patients with low and high ASNS mRNA expression.

The m6A writer METTL3 has been proved to be associated with cancer progression in a variety of cancer types (68). Here, we showed how to explore the function of METTL3 in liver cancer using RM2Target. TCGA data shows METTL3 expression is significantly higher in tumor tissues than normal tissues in LIHC (Figure 4A) and METTL3 expression is significantly associated with shorter overall survival in LIHC patients (Figure 4B). We next used RM2Target to find potential targets of METTL3 and its m6A effector in liver cancer to provide candidates for further functional and mechanism studies. Since IGF2BP1 has most target records among m6A readers in HepG2, a liver cancer cell line, we selected IGF2BP1 as the potential m6A effector in liver cancer. We observed a significant overlap between METTL3 target genes and IGF2BP1 target genes in HepG2 (Figure 4C). Moreover, there was also a large overlap between genes significantly correlated with METTL3 expression and genes significantly correlated with IGF2BP1 expression in TCGA LIHC gene expression data (Figure 4C). 485 genes were overlapped between RM2Target results and TCGA results, which are candidate target genes of both METTL3 and IGF2BP1 (Figure 4C). The 485 target genes were enriched in many cancer-related pathways, such as P53 signaling pathway and Apoptosis (Figure 4D). Next, we performed survival analysis using univariate Cox model and found 36 targets significantly associated with overall survival (Figure 4E). We then used RM2Target to browse the detailed information of these targets. We take ASNS (asparagine synthetase (glutamine-hydrolyzing)) as an example (http://rm2target.canceromics.org/#/detail/RM2Target_260294; http://rm2target.canceromics.org/#/detail/RM2Target_900943). We noted that ASNS was associated with cancer and malignant neoplasm of liver according to the disease annotations in RM2Target (Figure 4F). The expression level of ASNS was significantly downregulated upon METTL3 knockdown (Figure 4G), and the expression level of METTL3 and ASNS showed positive correlation in TCGA LIHC data (Figure 4H). The expression level of ASNS was significantly downregulated upon IGF2BP1 knockdown (Figure 4I), and the expression level of IGF2BP1 and ASNS showed positive correlation in TCGA LIHC data (Figure 4J). Additionally, ASNS harbours many m6A modification sites, and IGF2BP1 can bind to ASNS according to the POSTAR3 annotations collected in RM2Target. The high expression of ASNS was associated with poor prognosis according to the TCGA LIHC data (Figure 4K). Taken together, the above results suggest that ASNS may be a reliable target gene of METTL3/IGF2BP1, and METTL3/m6A(ASNS)/IGF2BP1 axis may serve as a novel promising therapeutic target for liver cancer, which deserved to be investigated in depth.

## SUMMARY AND PERSPECTIVES

Determining the regulatory network of WERs will be of great significance in the understanding of the molecular mechanisms underlying the regulation of RNA modifications and exploration of the potential application value of RNA modifications. To our knowledge, RM2Target is the first comprehensive and specific resources focusing on the targets of different WERs across various RNA modifications.

Our previous version m6A2Target received much attention and facilitated a lot of m6A related research. For examples, the researchers use m6A2Target to explore the tumorigenic mechanisms of m6A WERs in various cancer types (68,69). Compared to m6A2Target, RM2Target covers more types of RNA modifications and collects more WER–target gene associations which expands nearly 4 times more than m6A2Target. Using RM2Target to retrieve target genes for WERs of interest can help researchers conveniently and quickly identify the candidate genes, further promote the study of regulatory mechanisms. Moreover, researchers can explore the potential functions of RNA modifications with the perspective of crosstalk between different WERs and between different RNA modifications as mentioned above. However, RM2Target also has limitations. First, we only collected data from the two most frequently used organisms: human and mouse. Second, the target gene type and evidence type are not comprehensive enough. Currently, RM2Target does not provided WER–target associations for the target gene types such as circRNA and small RNAs. For the perturbation targets, we only focused on the five most studied downstream effects such as expression, modification, translation, stability, and alternative splicing. In addition, the targets were mainly derived from short read sequencing data. Long reads sequencing technology is a new trend for modification detection, which is not included in the current version. We will attempt to fix the above limitations in the future updates of RM2Target.

In conclusion, we expect that RM2Target will become a powerful resource for the field of RNA modification research. As a long-term goal, we would like to continually update RM2Target.

## DATA AVAILABILITY

RM2Target is a comprehensive online database available at http://rm2target.canceromics.org.

## Supplementary Material

gkac945_Supplemental_Files

## References

[1] Roundtree I.A. , Evans M.E., Pan T., He C. Dynamic RNA modifications in gene expression regulation. Cell. 2017; 169:1187–1200. 28622506 10.1016/j.cell.2017.05.045 PMC5657247

[2] Liu J. , Yue Y., Han D., Wang X., Fu Y., Zhang L., Jia G., Yu M., Lu Z., Deng X. et al. A METTL3-METTL14 complex mediates mammalian nuclear RNA N6-adenosine methylation. Nat. Chem. Biol. 2014; 10:93–95. 24316715 10.1038/nchembio.1432 PMC3911877

[3] Yang X. , Yang Y., Sun B.F., Chen Y.S., Xu J.W., Lai W.Y., Li A., Wang X., Bhattarai D.P., Xiao W. et al. 5-methylcytosine promotes mRNA export - NSUN2 as the methyltransferase and ALYREF as an m(5)C reader. Cell Res. 2017; 27:606–625. 28418038 10.1038/cr.2017.55 PMC5594206

[4] Jia G. , Fu Y., Zhao X., Dai Q., Zheng G., Yang Y., Yi C., Lindahl T., Pan T., Yang Y.G. et al. N6-methyladenosine in nuclear RNA is a major substrate of the obesity-associated FTO. Nat. Chem. Biol. 2011; 7:885–887. 22002720 10.1038/nchembio.687 PMC3218240

[5] Chen Z. , Qi M., Shen B., Luo G., Wu Y., Li J., Lu Z., Zheng Z., Dai Q., Wang H. Transfer RNA demethylase ALKBH3 promotes cancer progression via induction of tRNA-derived small RNAs. Nucleic Acids Res. 2019; 47:2533–2545. 30541109 10.1093/nar/gky1250 PMC6411830

[6] Shi H. , Wang X., Lu Z., Zhao B.S., Ma H., Hsu P.J., Liu C., He C. YTHDF3 facilitates translation and decay of N(6)-methyladenosine-modified RNA. Cell Res. 2017; 27:315–328. 28106072 10.1038/cr.2017.15 PMC5339834

[7] Chang C.T. , Hautbergue G.M., Walsh M.J., Viphakone N., van Dijk T.B., Philipsen S., Wilson S.A. Chtop is a component of the dynamic TREX mRNA export complex. EMBO J. 2013; 32:473–486. 23299939 10.1038/emboj.2012.342 PMC3567497

[8] Barbieri I. , Kouzarides T. Role of RNA modifications in cancer. Nat. Rev. Cancer. 2020; 20:303–322. 32300195 10.1038/s41568-020-0253-2

[9] Wu S. , Zhang S., Wu X., Zhou X. m(6)A RNA methylation in cardiovascular diseases. Mol. Ther. 2020; 28:2111–2119. 32910911 10.1016/j.ymthe.2020.08.010 PMC7544996

[10] Jonkhout N. , Tran J., Smith M.A., Schonrock N., Mattick J.S., Novoa E.M. The RNA modification landscape in human disease. RNA. 2017; 23:1754–1769. 28855326 10.1261/rna.063503.117 PMC5688997

[11] Li T. , Hu P.S., Zuo Z., Lin J.F., Li X., Wu Q.N., Chen Z.H., Zeng Z.L., Wang F., Zheng J. et al. METTL3 facilitates tumor progression via an m(6)A-IGF2BP2-dependent mechanism in colorectal carcinoma. Mol. Cancer. 2019; 18:112. 31230592 10.1186/s12943-019-1038-7 PMC6589893

[12] Chen M. , Wei L., Law C.T., Tsang F.H., Shen J., Cheng C.L., Tsang L.H., Ho D.W., Chiu D.K., Lee J.M. et al. RNA N6-methyladenosine methyltransferase-like 3 promotes liver cancer progression through YTHDF2-dependent posttranscriptional silencing of SOCS2. Hepatology. 2018; 67:2254–2270. 29171881 10.1002/hep.29683

[13] Li X. , Tang J., Huang W., Wang F., Li P., Qin C., Qin Z., Zou Q., Wei J., Hua L. et al. The M6A methyltransferase METTL3: acting as a tumor suppressor in renal cell carcinoma. OncoTargets Ther. 2017; 8:96103–96116. 10.18632/oncotarget.21726 PMC5707084 29221190

[14] Lin J.S. , Lai E.M. Protein-Protein interactions: co-immunoprecipitation. Methods Mol. Biol. 2017; 1615:211–219. 28667615 10.1007/978-1-4939-7033-9_17

[15] Zambelli F. , Pavesi G. RIP-Seq data analysis to determine RNA–protein associations. Methods Mol. Biol. 2015; 1269:293–303. 25577386 10.1007/978-1-4939-2291-8_18

[16] Jankowsky E. , Harris M.E. Specificity and nonspecificity in RNA–protein interactions. Nat. Rev. Mol. Cell Biol. 2015; 16:533–544. 26285679 10.1038/nrm4032 PMC4744649

[17] Nikam R.R. , Gore K.R. Journey of siRNA: clinical developments and targeted delivery. Nucleic Acid Ther. 2018; 28:209–224. 29584585 10.1089/nat.2017.0715

[18] Cong L. , Ran F.A., Cox D., Lin S., Barretto R., Habib N., Hsu P.D., Wu X., Jiang W., Marraffini L.A. et al. Multiplex genome engineering using CRISPR/Cas systems. Science. 2013; 339:819–823. 23287718 10.1126/science.1231143 PMC3795411

[19] Deng S. , Zhang H., Zhu K., Li X., Ye Y., Li R., Liu X., Lin D., Zuo Z., Zheng J. M6A2Target: a comprehensive database for targets of m6A writers, erasers and readers. Brief Bioinform. 2021; 22:bbaa055. 32392583 10.1093/bib/bbaa055

[20] Barrett T. , Wilhite S.E., Ledoux P., Evangelista C., Kim I.F., Tomashevsky M., Marshall K.A., Phillippy K.H., Sherman P.M., Holko M. et al. NCBI GEO: archive for functional genomics data sets–update. Nucleic Acids Res. 2013; 41:D991–D995. 23193258 10.1093/nar/gks1193 PMC3531084

[21] Chen S. , Zhou Y., Chen Y., Gu J. fastp: an ultra-fast all-in-one FASTQ preprocessor. Bioinformatics. 2018; 34:i884–i890. 30423086 10.1093/bioinformatics/bty560 PMC6129281

[22] Langmead B. , Salzberg S.L. Fast gapped-read alignment with bowtie 2. Nat. Methods. 2012; 9:357–359. 22388286 10.1038/nmeth.1923 PMC3322381

[23] Thomas R. , Thomas S., Holloway A.K., Pollard K.S. Features that define the best chip-seq peak calling algorithms. Brief Bioinform. 2017; 18:441–450. 27169896 10.1093/bib/bbw035 PMC5429005

[24] Heinz S. , Benner C., Spann N., Bertolino E., Lin Y.C., Laslo P., Cheng J.X., Murre C., Singh H., Glass C.K. Simple combinations of lineage-determining transcription factors prime cis-regulatory elements required for macrophage and b cell identities. Mol. Cell. 2010; 38:576–589. 20513432 10.1016/j.molcel.2010.05.004 PMC2898526

[25] Dobin A. , Davis C.A., Schlesinger F., Drenkow J., Zaleski C., Jha S., Batut P., Chaisson M., Gingeras T.R. STAR: ultrafast universal RNA-seq aligner. Bioinformatics. 2013; 29:15–21. 23104886 10.1093/bioinformatics/bts635 PMC3530905

[26] Li B. , Dewey C.N. RSEM: accurate transcript quantification from RNA-Seq data with or without a reference genome. BMC Bioinf. 2011; 12:323. 10.1186/1471-2105-12-323 PMC3163565 21816040

[27] Shah A. , Qian Y., Weyn-Vanhentenryck S.M., Zhang C. CLIP tool kit (CTK): a flexible and robust pipeline to analyze CLIP sequencing data. Bioinformatics. 2017; 33:566–567. 27797762 10.1093/bioinformatics/btw653 PMC6041811

[28] Corcoran D.L. , Georgiev S., Mukherjee N., Gottwein E., Skalsky R.L., Keene J.D., Ohler U. PARalyzer: definition of RNA binding sites from PAR-CLIP short-read sequence data. Genome Biol. 2011; 12:R79. 21851591 10.1186/gb-2011-12-8-r79 PMC3302668

[29] Love M.I. , Huber W., Anders S. Moderated estimation of fold change and dispersion for RNA-seq data with DESeq2. Genome Biol. 2014; 15:550. 25516281 10.1186/s13059-014-0550-8 PMC4302049

[30] Bao X. , Zhu K., Liu X., Chen Z., Luo Z., Zhao Q., Ren J., Zuo Z. MeRIPseqPipe: an integrated analysis pipeline for merip-seq data based on nextflow. Bioinformatics. 2022; 38:2054–2056. 10.1093/bioinformatics/btac025 35022687

[31] Cui X. , Meng J., Zhang S., Chen Y., Huang Y. A novel algorithm for calling mRNA m6A peaks by modeling biological variances in merip-seq data. Bioinformatics. 2016; 32:i378–i385. 27307641 10.1093/bioinformatics/btw281 PMC4908365

[32] Chen C.Y. , Ezzeddine N., Shyu A.B. Messenger RNA half-life measurements in mammalian cells. Methods Enzymol. 2008; 448:335–357. 19111184 10.1016/S0076-6879(08)02617-7 PMC2778729

[33] Shen S. , Park J.W., Lu Z.X., Lin L., Henry M.D., Wu Y.N., Zhou Q., Xing Y. rMATS: robust and flexible detection of differential alternative splicing from replicate RNA-Seq data. Proc. Natl. Acad. Sci. U.S.A. 2014; 111:E5593–E5601. 25480548 10.1073/pnas.1419161111 PMC4280593

[34] Quinlan A.R. , Hall I.M. BEDTools: a flexible suite of utilities for comparing genomic features. Bioinformatics. 2010; 26:841–842. 20110278 10.1093/bioinformatics/btq033 PMC2832824

[35] Frankish A. , Diekhans M., Jungreis I., Lagarde J., Loveland J.E., Mudge J.M., Sisu C., Wright J.C., Armstrong J., Barnes I. et al. Gencode 2021. Nucleic Acids Res. 2021; 49:D916–D923. 33270111 10.1093/nar/gkaa1087 PMC7778937

[36] Howe K.L. , Achuthan P., Allen J., Allen J., Alvarez-Jarreta J., Amode M.R., Armean I.M., Azov A.G., Bennett R., Bhai J. et al. Ensembl 2021. Nucleic Acids Res. 2021; 49:D884–D891. 33137190 10.1093/nar/gkaa942 PMC7778975

[37] Navarro Gonzalez J. , Zweig A.S., Speir M.L., Schmelter D., Rosenbloom K.R., Raney B.J., Powell C.C., Nassar L.R., Maulding N.D., Lee C.M. et al. The UCSC genome browser database: 2021 update. Nucleic Acids Res. 2021; 49:D1046–d1057. 33221922 10.1093/nar/gkaa1070 PMC7779060

[38] Chan P.P. , Lowe T.M. GtRNAdb 2.0: an expanded database of transfer RNA genes identified in complete and draft genomes. Nucleic Acids Res. 2016; 44:D184–D189. 26673694 10.1093/nar/gkv1309 PMC4702915

[39] Luo X. , Li H., Liang J., Zhao Q., Xie Y., Ren J., Zuo Z. RMVar: an updated database of functional variants involved in RNA modifications. Nucleic Acids Res. 2021; 49:D1405–D1412. 33021671 10.1093/nar/gkaa811 PMC7779057

[40] Xuan J.J. , Sun W.J., Lin P.H., Zhou K.R., Liu S., Zheng L.L., Qu L.H., Yang J.H. RMBase v2.0: deciphering the map of RNA modifications from epitranscriptome sequencing data. Nucleic Acids Res. 2018; 46:D327–D334. 29040692 10.1093/nar/gkx934 PMC5753293

[41] Ma J. , Song B., Wei Z., Huang D., Zhang Y., Su J., de Magalhães J.P., Rigden D.J., Meng J., Chen K. m5C-Atlas: a comprehensive database for decoding and annotating the 5-methylcytosine (m5C) epitranscriptome. Nucleic Acids Res. 2022; 50:D196–D203. 34986603 10.1093/nar/gkab1075 PMC8728298

[42] Li J.H. , Liu S., Zhou H., Qu L.H., Yang J.H. starBase v2.0: decoding miRNA-ceRNA, miRNA-ncRNA and protein–RNA interaction networks from large-scale CLIP-Seq data. Nucleic Acids Res. 2014; 42:D92–D97. 24297251 10.1093/nar/gkt1248 PMC3964941

[43] Zhao W. , Zhang S., Zhu Y., Xi X., Bao P., Ma Z., Kapral T.H., Chen S., Zagrovic B., Yang Y.T. et al. POSTAR3: an updated platform for exploring post-transcriptional regulation coordinated by RNA-binding proteins. Nucleic Acids Res. 2022; 50:D287–D294. 34403477 10.1093/nar/gkab702 PMC8728292

[44] Teng X. , Chen X., Xue H., Tang Y., Zhang P., Kang Q., Hao Y., Chen R., Zhao Y., He S. NPInter v4.0: an integrated database of ncRNA interactions. Nucleic Acids Res. 2020; 48:D160–D165. 31670377 10.1093/nar/gkz969 PMC7145607

[45] Cancer Genome Atlas Research, N. Weinstein J.N. , Collisson E.A., Mills G.B., Shaw K.R., Ozenberger B.A., Ellrott K., Shmulevich I., Sander C., Stuart J.M The cancer genome atlas pan-cancer analysis project. Nat. Genet. 2013; 45:1113–1120. 24071849 10.1038/ng.2764 PMC3919969

[46] Amberger J.S. , Bocchini C.A., Scott A.F., Hamosh A. OMIM.org: leveraging knowledge across phenotype-gene relationships. Nucleic Acids Res. 2019; 47:D1038–D1043. 30445645 10.1093/nar/gky1151 PMC6323937

[47] Pinero J. , Ramirez-Anguita J.M., Sauch-Pitarch J., Ronzano F., Centeno E., Sanz F., Furlong L.I. The DisGeNET knowledge platform for disease genomics: 2019 update. Nucleic Acids Res. 2020; 48:D845–D855. 31680165 10.1093/nar/gkz1021 PMC7145631

[48] Ning L. , Cui T., Zheng B., Wang N., Luo J., Yang B., Du M., Cheng J., Dou Y., Wang D 2021) MNDR v3.0: mammal ncRNA–disease repository with increased coverage and annotation. Nucleic Acids Res. 49:D160–D164. 32833025 10.1093/nar/gkaa707 PMC7779040

[49] Bao Z. , Yang Z., Huang Z., Zhou Y., Cui Q., Dong D LncRNADisease 2.0: an updated database of long non-coding RNA-associated diseases. Nucleic Acids Res. 2019; 47:D1034–D1037. 30285109 10.1093/nar/gky905 PMC6324086

[50] Xia S. , Feng J., Chen K., Ma Y., Gong J., Cai F., Jin Y., Gao Y., Xia L., Chang H. et al. CSCD: a database for cancer-specific circular RNAs. Nucleic Acids Res. 2018; 46:D925–D929. 29036403 10.1093/nar/gkx863 PMC5753219

[51] Landrum M.J. , Lee J.M., Benson M., Brown G., Chao C., Chitipiralla S., Gu B., Hart J., Hoffman D., Hoover J. et al. ClinVar: public archive of interpretations of clinically relevant variants. Nucleic Acids Res. 2016; 44:D862–D868. 26582918 10.1093/nar/gkv1222 PMC4702865

[52] Tate J.G. , Bamford S., Jubb H.C., Sondka Z., Beare D.M., Bindal N., Boutselakis H., Cole C.G., Creatore C., Dawson E. et al. COSMIC: the catalogue of somatic mutations in cancer. Nucleic Acids Res. 2019; 47:D941–D947. 30371878 10.1093/nar/gky1015 PMC6323903

[53] Zhang J. , Bajari R., Andric D., Gerthoffert F., Lepsa A., Nahal-Bose H., Stein L.D., Ferretti V. The international cancer genome consortium data portal. Nat. Biotechnol. 2019; 37:367–369. 30877282 10.1038/s41587-019-0055-9

[54] Huang H. , Weng H., Zhou K., Wu T., Zhao B.S., Sun M., Chen Z., Deng X., Xiao G., Auer F. et al. Histone H3 trimethylation at lysine 36 guides m(6)A RNA modification co-transcriptionally. Nature. 2019; 567:414–419. 30867593 10.1038/s41586-019-1016-7 PMC6438714

[55] Barbieri I. , Tzelepis K., Pandolfini L., Shi J., Millán-Zambrano G., Robson S.C., Aspris D., Migliori V., Bannister A.J., Han N. et al. Promoter-bound METTL3 maintains myeloid leukaemia by m(6)A-dependent translation control. Nature. 2017; 552:126–131. 29186125 10.1038/nature24678 PMC6217924

[56] Xu W. , He C., Kaye E.G., Li J., Mu M., Nelson G.M., Dong L., Wang J., Wu F., Shi Y.G. et al. Dynamic control of chromatin-associated m(6)A methylation regulates nascent RNA synthesis. Mol. Cell. 2022; 82:1156–1168. 35219383 10.1016/j.molcel.2022.02.006 PMC8969783

[57] Wang X. , Lu Z., Gomez A., Hon G.C., Yue Y., Han D., Fu Y., Parisien M., Dai Q., Jia G. et al. N6-methyladenosine-dependent regulation of messenger RNA stability. Nature. 2014; 505:117–120. 24284625 10.1038/nature12730 PMC3877715

[58] Mendel M. , Delaney K., Pandey R.R., Chen K.M., Wenda J.M., Vagbo C.B., Steiner F.A., Homolka D., Pillai R.S. Splice site m(6)A methylation prevents binding of U2AF35 to inhibit RNA splicing. Cell. 2021; 184:3125–3142. 33930289 10.1016/j.cell.2021.03.062 PMC8208822

[59] Shi H. , Chai P., Jia R., Fan X. Novel insight into the regulatory roles of diverse RNA modifications: Re-defining the bridge between transcription and translation. Mol. Cancer. 2020; 19:78. 32303268 10.1186/s12943-020-01194-6 PMC7164178

[60] Panneerdoss S. , Eedunuri V.K., Yadav P., Timilsina S., Rajamanickam S., Viswanadhapalli S., Abdelfattah N., Onyeagucha B.C., Cui X., Lai Z. et al. Cross-talk among writers, readers, and erasers of m(6)A regulates cancer growth and progression. Sci. Adv. 2018; 4:eaar8263. 30306128 10.1126/sciadv.aar8263 PMC6170038

[61] Courtney D.G. , Chalem A., Bogerd H.P., Law B.A., Kennedy E.M., Holley C.L., Cullen B.R. Extensive epitranscriptomic methylation of a and c residues on murine leukemia virus transcripts enhances viral gene expression. Mbio. 2019; 10:e01209-19. 31186331 10.1128/mBio.01209-19 PMC6561033

[62] Xiang J.F. , Yang Q., Liu C.X., Wu M., Chen L.L., Yang L. N(6)-Methyladenosines modulate A-to-I RNA editing. Mol. Cell. 2018; 69:126–135. 29304330 10.1016/j.molcel.2017.12.006

[63] Qin W. , Lv P., Fan X., Quan B., Zhu Y., Qin K., Chen Y., Wang C., Chen X. Quantitative time-resolved chemoproteomics reveals that stable O-GlcNAc regulates box C/D snoRNP biogenesis. Proc. Natl. Acad. Sci. U.S.A. 2017; 114:E6749–E6758. 28760965 10.1073/pnas.1702688114 PMC5565422

[64] Li Y. , Xia L., Tan K., Ye X., Zuo Z., Li M., Xiao R., Wang Z., Liu X., Deng M. et al. N(6)-Methyladenosine co-transcriptionally directs the demethylation of histone H3K9me2. Nat. Genet. 2020; 52:870–877. 32778823 10.1038/s41588-020-0677-3

[65] Liu J. , Dou X., Chen C., Chen C., Liu C., Xu M.M., Zhao S., Shen B., Gao Y., Han D. et al. N (6)-methyladenosine of chromosome-associated regulatory RNA regulates chromatin state and transcription. Science. 2020; 367:580–586. 31949099 10.1126/science.aay6018 PMC7213019

[66] Wei X. , Huo Y., Pi J., Gao Y., Rao S., He M., Wei Q., Song P., Chen Y., Lu D. et al. METTL3 preferentially enhances non-m(6)A translation of epigenetic factors and promotes tumourigenesis. Nat. Cell Biol. 2022; 24:1278–1290. 35927451 10.1038/s41556-022-00968-y

[67] Su R. , Dong L., Li Y., Gao M., He P.C., Liu W., Wei J., Zhao Z., Gao L., Han L. et al. METTL16 exerts an m(6)A-independent function to facilitate translation and tumorigenesis. Nat. Cell Biol. 2022; 24:205–216. 35145225 10.1038/s41556-021-00835-2 PMC9070413

[68] Zhu Y. , Peng X., Zhou Q., Tan L., Zhang C., Lin S., Long M. METTL3-mediated m6A modification targeting of STEAP2 mRNAinhibits papillary thyroid cancer progress by blocking the Hedgehog signalingpathway and epithelial-to-mesenchymal transition. Cell Death Dis. 2022; 13:358. 35436987 10.1038/s41419-022-04817-6 PMC9016063

[69] Wan W. , Ao X., Chen Q., Yu Y., Ao L., Xing W., Guo W., Wu X., Pu C., Hu X. et al. METTL3/IGF2BP3 axis inhibits tumor immune surveillance by upregulating N^6^-methyladenosine modification of PD-L1 mRNA in breast cancer. Mol. Cancer. 2022; 21:60. 35197058 10.1186/s12943-021-01447-y PMC8864846

