# Supplementary material for: RM2Target: a comprehensive database for targets of writers, erasers and readers of RNA modifications
Source: Nucleic Acids Res. 2022 Oct 27;51(D1):D269–79. doi: 10.1093/nar/gkac945 (PMC9825529; doi:10.1093/nar/gkac945)
Supplement: gkac945_Supplemental_Files [file gkac945_supplemental_files.zip › Supplementary_materials-.pdf]

SUPPLEMENTARY MATERIALS

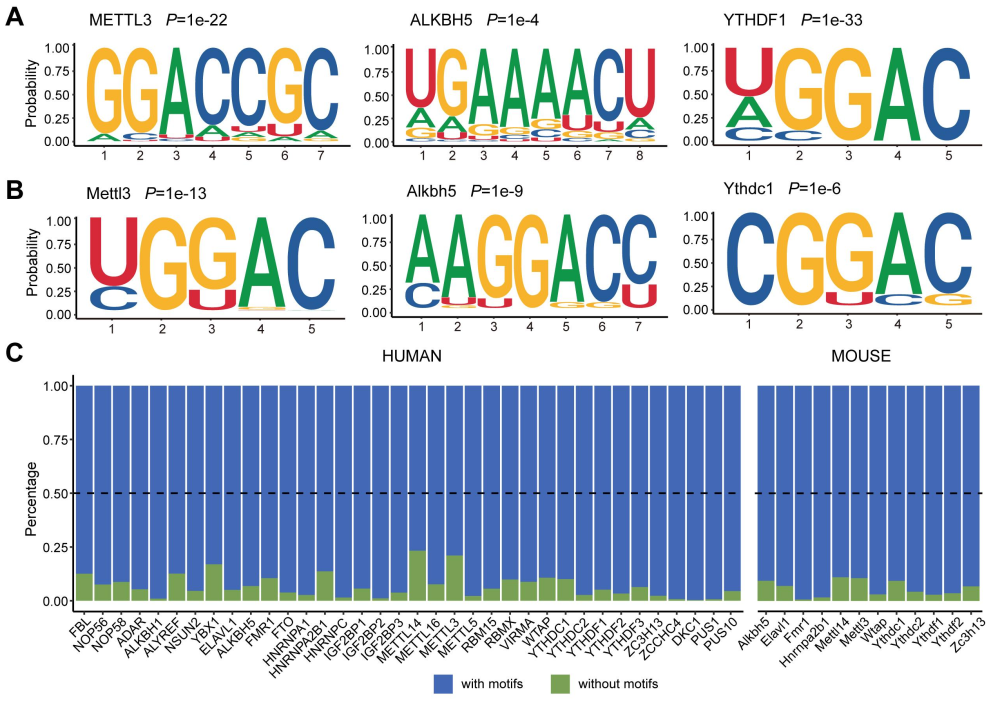

Figure S1. Motif analysis of target genes for individual WERs. (A) m6A consensus motifs detected in human WER targets. (B) m6A consensus motifs detected in mouse WER targets. (C) The proportion of target genes with or without corresponding motifs.
